# Oligoprogressive Non-Small-Cell Lung Cancer under Treatment with PD-(L)1 Inhibitors

**DOI:** 10.3390/cancers12041046

**Published:** 2020-04-23

**Authors:** Stephan Rheinheimer, Claus-Peter Heussel, Philipp Mayer, Lena Gaissmaier, Farastuk Bozorgmehr, Hauke Winter, Felix J. Herth, Thomas Muley, Stephan Liersch, Helge Bischoff, Mark Kriegsmann, Rami A. El Shafie, Albrecht Stenzinger, Michael Thomas, Hans-Ulrich Kauczor, Petros Christopoulos

**Affiliations:** 1 Translational Lung Research Center Heidelberg (TLRC-H), Member of the German Center for Lung Research (DZL), 69120 Heidelberg, Germany; Philipp.Mayer@med.uni-heidelberg.de (P.M.); Farastuk.Bozorgmehr@med.uni-heidelberg.de (F.B.); Hauke.Winter@med.uni-heidelberg.de (H.W.); Felix.Herth@med.uni-heidelberg.de (F.J.H.); Thomas.Muley@med.uni-heidelberg.de (T.M.); Helge.Bischoff@med.uni-heidelberg.de (H.B.); Mark.Kriegsmann@med.uni-heidelberg.de (M.K.); Albrecht.Stenzinger@med.uni-heidelberg.de (A.S.); Michael.Thomas@med.uni-heidelberg.de (M.T.); Hans-Ulrich.Kauczor@med.uni-heidelberg.de (H.-U.K.); 2 Department for Diagnostic and Interventional Radiology, Heidelberg University Hospital, 69120 Heidelberg, Germany; 3 Diagnostic and Interventional Radiology with Nuclear Medicine, Thoraxklinik at Heidelberg University Hospital, 69126 Heidelberg, Germany; 4 Department of Thoracic Oncology, Thoraxklinik at Heidelberg University Hospital, 69126 Heidelberg, Germany; Lena.Gaissmaier@med.uni-heidelberg.de; 5 Department of Thoracic Surgery, Thoraxklinik at Heidelberg University Hospital, 69126 Heidelberg, Germany; 6 Department of Pneumology, Thoraxklinik at Heidelberg University Hospital, 69126 Heidelberg, Germany; 7 Translational Research Unit, Thoraxklinik at Heidelberg University Hospital, 69126 Heidelberg, Germany; 8 Pharmacy, Thoraxklinik at Heidelberg University Hospital, 69126 Heidelberg, Germany; Stephan.Liersch@med.uni-heidelberg.de; 9 Institute of Pathology, Heidelberg University Hospital, 69120 Heidelberg, Germany; 10 Department of Radiation Oncology, Heidelberg University Hospital, 69120 Heidelberg, Germany; Rami.ElShafie@med.uni-heidelberg.de

**Keywords:** non-small-cell lung cancer, oligoprogression, immunotherapy, chemoimmunotherapy, local therapy

## Abstract

Oligoprogression (OPD) of non-small-cell lung cancer (NSCLC) occurs in approximately half of patients under targeted compounds (TKI) and facilitates use of regional therapies that can prolong survival. In order to characterize OPD in immunotherapy (IO)-treated NSCLC, we analyzed the failure pattern under PD-1/PD-L1 inhibitors (*n* = 297) or chemoimmunotherapy (*n* = 75). Under IO monotherapy, OPD was more frequent (20% vs. 10%, *p* < 0.05), occurred later (median 11 vs. 5 months, *p* < 0.01), affected fewer sites (mean 1.1 vs. 1.5, *p* < 0.05), and involved fewer lesions (1.4 vs. 2.3, *p* < 0.05) in the first compared to later lines. Lymph nodes (42%, mainly mediastinal) and the brain (39%) were mostly affected, followed by the lung (24%) and other organs. Compared to multifocal progression, OPD occurred later (11 vs. 4 months, *p* < 0.001) and was associated with longer survival (26 vs. 13 months, *p* < 0.001) and higher tumor PD-L1 expression (*p* < 0.001). Chemoimmunotherapy showed a similar incidence of OPD as IO monotherapy (13% vs. 11% at 2 years). Local treatments were applied regularly for brain but only in 50% for extracranial lesions. Thus, NSCLC oligoprogression is less common under IO than under TKI, but also favorable. Since its frequency drops later in the disease, regular restaging and multidisciplinary evaluation are essential in order to exploit the full therapeutic potential.

## 1. Introduction

Oligoprogression (OPD) is a relatively new concept that emerged as more effective systemic therapies became available in oncology and denotes anatomically restricted tumor progression in patients with otherwise controlled widespread disease [1]. Such an asynchronous behavior of metastatic disease has been observed in several tumor types, including melanoma, renal-cell, prostatic, and non-small-cell lung cancer (NSCLC) [1,2]. Its pathogenesis is considered to be complex, as numerous parameters, including molecular evolution of cancer cells, changes in the tumor microenvironment, hemodynamic parameters, and previous application of local therapies can potentially modulate the anatomical pattern of treatment failure. Notwithstanding, from a therapeutic point of view, OPD carries a very simple and important implication: the opportunity to regain control of disseminated tumors by use of local treatments, which can thereby prolong benefit from systemic therapies and patient survival [3]. Oncogene-driven NSCLC has been a model disease for the study of OPD, proper management of which has resulted in a median time-to-next-treatment gain of 5–10 months [3,4,5,6,7,8] and substantial overall survival (OS) improvement in several series [6,7]. In the particular context of immunotherapy (IO)-treated NSCLC, which includes the majority of lung cancer patients currently, smoldering tumor escape is not uncommon, but studies of OPD are presently lacking. The aim of this study was to characterize the frequency, radiologic characteristics, and potential clinical relevance of OPD in NSCLC patients under immunotherapy.

## 2. Results

**Figure 1:**
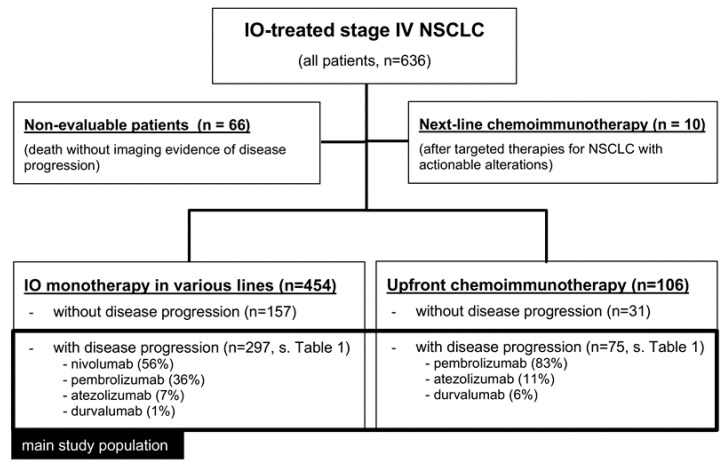
CONSORT diagram of the study.

**Figure 2:**
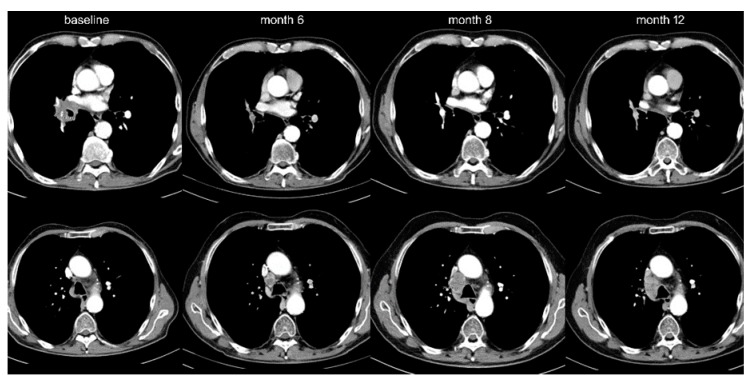
Lymph node oligoprogression. A 66-year-old male patient with adeno-NSCLC (PD-L1 90%) was started on pembrolizumab in November 2017. Nodal progression on the right side was noted in June 2018, which appeared stable in a subsequent restaging in October 2018, even though no change in therapy occurred.

**Figure 3:**
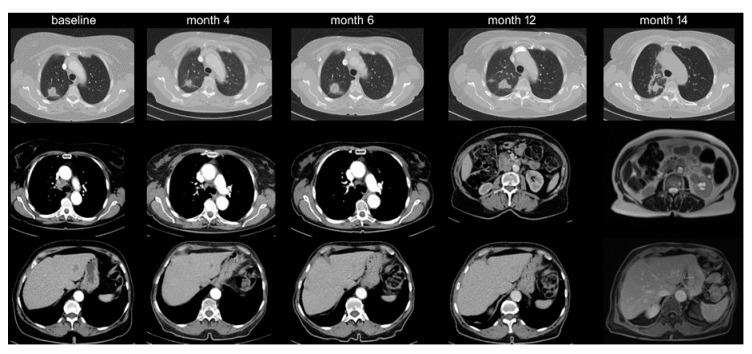
Lung oligoprogression and transitional cell carcinoma of the kidney. A 75-year-old female with adeno-NSCLC (PD-L1 90%) was started on pembrolizumab in September 2017 with response of the primary tumor, mediastinal lymph nodes, and liver metastases. Upon oligoprogression of the primary tumor in March 2018, thoracic radiotherapy was administered. In August 2018, a new kidney lesion was noted that grew oligoprogressive-like. At biopsy, this lesion turned out to be a transitional-cell carcinoma.

Among IO-monotherapy patients, 38 (13%) showed OPD with decreasing frequency beyond the first line (20% vs. 10%, *p* < 0.05, Table 1, Figure 1). Furthermore, OPD in patients treated with IO monotherapy in the first line occurred later (after 11 vs. 2 months in median, *p* < 0.001), involved fewer anatomical sites (mean 1.1 vs. 1.5, *p* < 0.05), and affected fewer lesions (mean 1.4 vs. 2.3, *p* < 0.05) compared to OPD in patients receiving IO monotherapy in later lines (Table 2). Lymph nodes (42% of OPD cases, mainly mediastinal, Figure 2 and Table 2) and brain (39%) were affected most frequently, but OPD was also observed in other organs typically affected by NSCLC, namely lung (24%, Figure 3), adrenal glands (16%), bone (8%), liver (5%), skin and soft tissues (3%).

**Figure 4:**
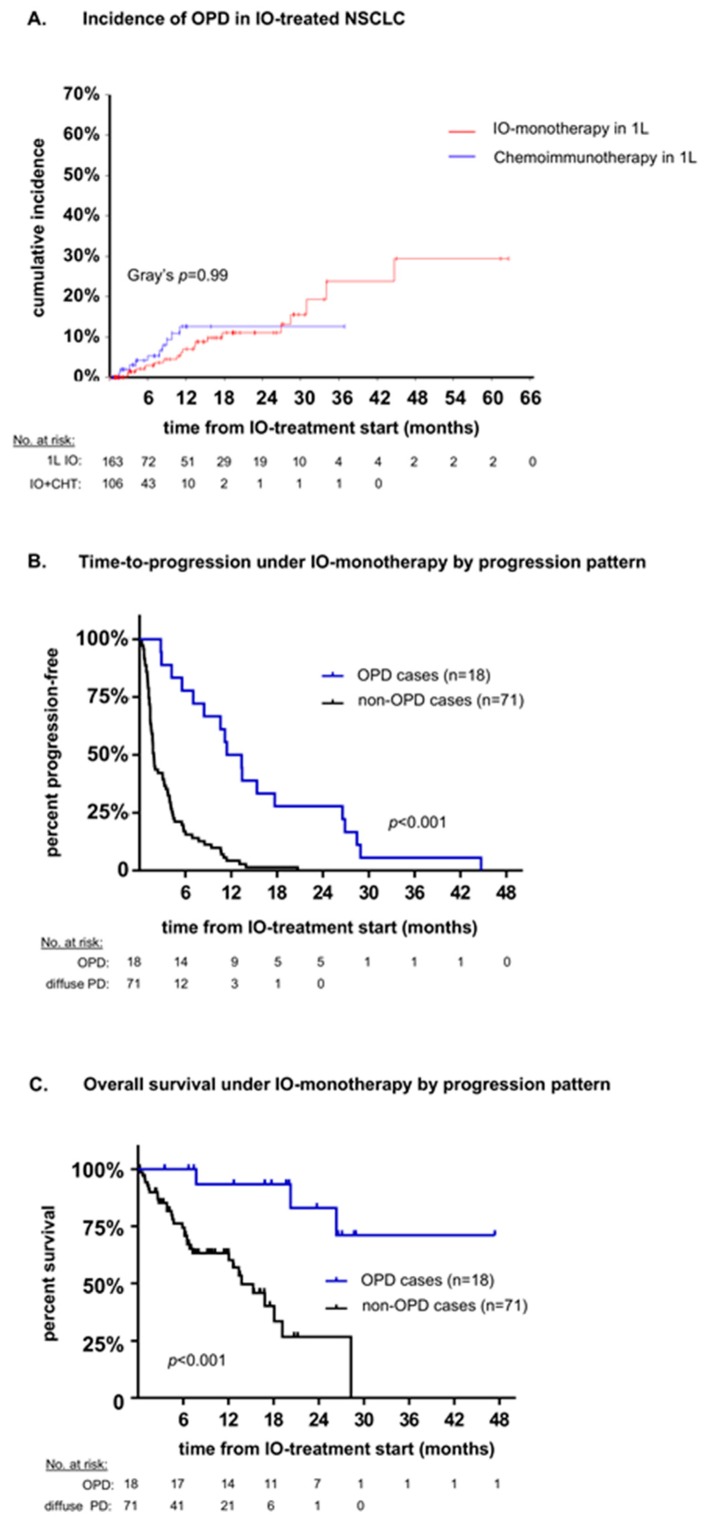
Incidence and prognosis of oligoprogression during first-line immunotherapy (to the right). (**A**) Cumulative incidence of oligoprogression (OPD) in stage IV NSCLC under first-line IO monotherapy (*n* = 163) vs. first-line chemoimmunotherapy (*n* = 106) in the entire study population (Figure 1). Patients without disease progression were censored, while OPD and diffuse progression were considered as competing risks. Cumulative incidence at 2 years was 12.6% for chemoimmunotherapy-treated vs. 11.0% for IO-monotherapy-treated patients (Gray’s *p* = 0.99). (**B**) Time-to-progression for NSCLC patients developing OPD (*n* = 18) vs. diffuse disease progression (*n* = 71) under first-line IO monotherapy (Table 1, log-rank *p* < 0.001). (**C**) Overall survival (OS) of NSCLC patients developing OPD (*n* = 18) vs. diffuse disease progression (*n* = 71), under first-line IO monotherapy (Table 1, log-rank *p* < 0.001).

The anatomic distribution of OPD was roughly similar across treatment lines, and the time to progression (TTP) for development of OPD did not differ significantly according to the organ involved (Table 2). NSCLC patients treated with first-line chemoimmunotherapy showed a similar incidence of oligoprogression as patients treated with first-line IO monotherapy (Figure 4A). Of note, the follow-up of chemoimmunotherapy patients in our study is shorter than that of IO monotherapy patients (7 vs. 15 months in median, Table 2), because chemoimmunotherapy was approved more recently for the treatment of non-squamous (September 2018) and squamous (March 2019) NSCLC in Europe.

OPD among IO-monotherapy patients was associated with a higher tumor PD-L1 expression (mean 65% vs. 41%, *p* < 0.001) than multifocal progression and was not encountered in any never-smokers in our cohort (*p* < 0.05), but other clinical characteristics of OPD and diffusely progressive patients were very similar (Table 1). No factor associated with OPD could be identified in chemoimmunotherapy-treated patients (Table 1). The characteristics of all OPD cases in our study are given in Table S1. The association between PD-L1 expression and OPD remained significant also in a multivariable analysis including all patient characteristics (Table S2).

Upon histologic verification, two patients (5%) with suspected OPD were found to harbor a second primary tumor, namely a hepatocellular carcinoma and a transitional cell carcinoma of the kidney (Figure 3).

### 2.1. Association of OPD with Patient Survival

OPD occurred later after start of IO monotherapy (median TTP 9 vs. 2 months, *p* < 0.001, Table 1 and Figure 4B) and was associated with longer overall survival than multifocal progression (mean 32 vs. 16 months, *p* < 0.001, Table 1 and Figure 4C). In a multivariable analysis based on all patient characteristics included in this study, the pattern of disease progression, i.e., OPD vs. diffuse, showed the strongest association with overall survival from IO treatment start (Table S3). Median time to first subsequent progression (TFSP, observed in 28/38 OPD cases) was 14 months (range 1–26 months), with comparable frequencies of multifocal tumor growth (15/28 = 54%) and “tandem OPD” (13/28 = 46%). For chemoimmunotherapy-treated patients, analysis of TTP according to the pattern of initial progression, as well as analysis of TFSP, were limited by the relatively short follow-up and low number of subsequent progression events (only 1/10 chemoimmunotherapy-treated OPD patients had developed subsequent progression up to the time of this study, and this was a “tandem OPD”).

### 2.2. Therapeutic Relevance of Oligoprogression

Local treatments were offered to 19/38 (50%) of IO-monotherapy- and 9/10 (90%) of chemoimmunotherapy-treated patients in our study. Across both patient subgroups, local therapies were more frequently applied to patients with cerebral OPD, 11/16 (72%) of which received brain radiotherapy, with the exceptions being either asymptomatic (*n* = 2) or having already been cerebrally irradiated in the past (*n* = 3). In contrast, the rate of local therapies was lower in case of extracerebral OPD (18/37 or 49%, 16/18 with radiotherapy and 2/18 with surgery). Based on radiological and clinical features, an additional 12/37 (32%) cases with extracerebral OPD could also have received local therapies, resulting in a total rate of eligibility comparable to that of brain OPD (30/37 or 81%).

## 3. Discussion

The clinical features of oligoprogression in oncogene-driven NSCLC have been well characterized in several studies [3,4,5,6,7,8,9,10]. For example, Yu et al. analyzed a group of 184 EGFR patients failing erlotinib or gefitinib and found an OPD rate of 33% with an intra- to extracranial ratio of about 2:1 (23% vs. 10%) [8]. Similar observations were made by several other investigators, for example, Weickhardt et al., in a cohort of 51 EGFR or ALK-mutated patients under erlotinib or crizotinib, 35% and 14% of which developed brain and extracranial OPD, respectively [5]. In contrast, brain was outnumbered by other OPD sites among 50 EGFR T790M-positive patients treated with osimertinib, even though the overall OPD rate was even higher at 72% [4]. Apparently, the brain efficacy of the administered compound, which is very high and similar to the systemic efficacy for osimertinib, but lower for erlotinib, gefitinib, and crizotinib, influences the balance between intra- and extracranial oligoprogression [11]. Immunotherapy is characterized by comparable intra- and extracranial efficacies [12], which is consistent with the predominance of non-brain OPD observed in our patients (Table 2), similar to the osimertinib study [4]. Another conclusion is that the OPD rate in NSCLC under targeted therapies is high and varies between 30%–70%, depending on operational definition, baseline patient status, type of therapy, frequency, and duration of follow-up [1,13].

In our study, the frequency of OPD among NSCLC patients receiving either IO monotherapy or chemoimmunotherapy was lower, at about 10%–20% (Table 2). This is in line with the result of a smaller series including 81 immunotherapy-treated patients that detected a rate of 11% (9/81) for OPD using slightly different criteria [14]. The lower rate of OPD in IO-treated NSCLC could be related to its presumably higher genetic instability, as inferred by the higher tumor mutational burden (TMB) [15,16], and to the lower efficacy of current immunotherapies compared to molecularly targeted drugs [17,18,19,20]. Interestingly, our results suggest similar OPD rates for first-line IO monotherapy compared to first-line chemoimmunotherapy (Figure 4A), which parallels the roughly similar efficacy of these treatments in terms of response rates and overall survival in the respective clinical studies, especially for patients with a higher PD-L1 expression, who are also more likely to develop OPD [19,20]. However, a special effect of chemoimmunotherapy is that it blunts the predictive significance of biomarkers relevant for IO monotherapy; for example, a higher PD-L1 expression, a higher TMB, and presence of *KRAS* mutations predict a better outcome of NSCLC patients treated with IO monotherapy but not chemoimmunotherapy [21,22,23,24]. This is likely due to an increased benefit of IO-unfavorable, i.e., PD-L1-negative, TMB-low, and *KRAS*-wild-type tumors from the additional chemotherapy. Along the same lines, in our study, PD-L1 expression was associated with OPD under IO monotherapy but showed no predictive value in case of chemoimmunotherapy (Table 1). Of note, the association of PD-L1 expression with OPD under IO monotherapy does not mean that PD-L1 is an accurate OPD biomarker, because we observed several (7/33 evaluable or 21%) IO-monotherapy-treated OPD cases with low (0–10%) PD-L1 expression (Table S1), and, conversely, many patients with high PD-L1 expression developed diffuse progression instead (Table 1). Furthermore, the blood lymphocyte-to-neutrophil ratio, a biomarker associated with efficacy of immunotherapy and chemotherapy in NSCLC [25,26], did not correlate with the pattern of NSCLC progression in our patients (Table 1 and Table S2). Overall, our results show that the development of OPD in IO-treated NSCLC cannot be reliably predicted based on commonly available clinical, laboratory, and pathologic parameters, which underlines the importance of radiologic surveillance for its detection. This is not surprising, since reliable biomarkers are currently not available for IO efficacy in general, and the development of OPD under PD-(L)1 inhibitors in a way represents greater benefit from immunotherapy in a special patient subset.

Indeed, the later occurrence of OPD compared to multifocal progression, its association with longer overall survival, and the occurrence of “tandem OPD” in about 50% of cases suggest a better effect of treatment and a more favorable biology of oligoprogressive NSCLC tumors. This changes during later disease stages, as tumors acquire more mutations, increase their heterogeneity, and become more aggressive [27,28]. This biologic deterioration of the tumors along with the decreased efficacy of immunotherapy in pretreated patients [29] is consistent with the lower frequency of OPD (Table 2), with the shorter time-to-OPD (Table 1), and with the shorter survival of OPD patients vs. patients with multifocal progression (Table 1) observed beyond the first line in our study. In keeping with this, the association of OPD with a higher PD-L1 expression (Table 1) suggests that immunologic tumor control is important for spatial containment of treatment failure, since PD-L1 expression in non-*EGFR/ALK* mutated NSCLC is associated with the presence of tumor-infiltrating lymphocytes and active anti-cancer immunity [30,31].

Enhanced immune activity in lymph nodes and lung has been proposed as the cause for better tumor control in these tissues compared to liver, adrenal glands, and bone in a study analyzing organ-specific response patterns in IO-treated NSCLC [32]. Since lymph nodes and lung are also very frequent sites of metastatic involvement in NSCLC, it is not surprising that they showed a higher frequency of OPD in our series (Table 2). On the other hand, liver OPD was rare in our patients (Table 2), which parallels the rarity of hepatic oligoprogression in EGFR-mutated NSCLC [4,5] and is in line with the lower immune reactivity and worse prognosis of hepatic metastases and hepatic oligoprogression in NSCLC [33,34,35].

From a therapeutic point of view, OPD is very important because it offers the opportunity to eradicate resistant tumor cell clones using local therapies [36], such as irradiation, surgery, or any percutaneous ablation, which can prolong survival [4]. In our patients, local treatments were generally offered to almost all brain OPD, but only half of extracranial OPD cases. Our retrospective analysis shows that the great majority of patients with extracranial OPD are also eligible for local therapies. Precise knowledge about the clinical and radiologic characteristics of OPD under immunotherapy, as elaborated by the present study, could enhance awareness for this special constellation in IO-treated NSCLC. Since the frequency of OPD drops later in the disease course, regular complete restaging under immunotherapy and multidisciplinary evaluation of new or enlarging lesions are of key importance in order to exploit the full therapeutic potential. The fact that up to half of oligoprogressive patients subsequently develop a second OPD underscores the importance of proper initial management, since such cases could potentially derive an even larger benefit through tandem use of local therapies. On the other hand, it is also imperative to verify ambiguous cases histologically, as some lesions might turn out to represent inflammatory reactions to immunotherapy [37,38] or even second primary tumors, as noted for two of our patients.

The main limitation of our study is its retrospective nature. In addition, histologic verification was not obtained for all OPD lesions, and their classification as such in many cases relied on clinical criteria including further progression of the respective lesions in follow-up imaging and clinical deterioration of the patient during the subsequent clinical course. Furthermore, due to the relatively recent approval of chemoimmunotherapy for NSCLC in Europe, the follow-up of our chemoimmunotherapy-treated patients is not very long.

## 4. Materials and Methods

The main study population encompassed all 372 stage IV NSCLC patients with radiologically documented disease progression under PD-1/PD-L1 inhibitors among all 636 IO-treated patients in our hospital from March 2013 until September 2019. Treatment consisted of either checkpoint inhibitor monotherapy in various treatment lines (454 IO-monotherapy patients, of which 297 had radiologic evidence of disease progression at the time of study inclusion) or upfront combined chemoimmunotherapy (106 patients, 75 of which with radiologic progression, Figure 1). For analysis, the study population was divided in three groups: patients with IO monotherapy in the first line (*n* = 89), who were contrasted to patients with IO monotherapy in subsequent treatment lines (*n* = 208) and to patients with chemoimmunotherapy in the first line (*n* = 75, Table 1). Imaging studies were performed according to the standard in our institution with chest/abdomen CT and brain MRI scans before treatment start and every 6–12 weeks thereafter (Figure 2). Cases with disease progression suitable for inclusion in this study were identified by a retrospective review of medical records, followed by a review of radiological images in order to characterize the progression pattern as diffuse or OPD. Other clinical parameters were collected from the patients’ records (Table 1). Patients with early death before the first restaging, and patients who died during the stable phase of the disease after the initial response due to other reasons, were non-evaluable and excluded from this analysis (*n* = 66). Patients with chemoimmunotherapy after pretreatment with tyrosine kinase inhibitors for actionable genetic alterations were also excluded from this study because of potential biologic differences (*n* = 10, Figure 1). Testing for actionable EGFR/ALK/ROS1/BRAF mutations had been performed as indicated (e.g., in case of adenocarcinomatous histology or never/light-smoker status) for all patients at initial diagnosis, followed by administration of targeted therapies if positive, so that practically no cases with treatable genetic alterations were included in this analysis.

Image analyses were performed at a routine PACS station by expert radiologists in cooperation with thoracic oncologists. OPD was defined as localized treatment failure at one or two anatomic sites, with one to five progressive measurable (according to RECIST 1.1) lesions, either new or with ≥ 20% growth of their longest diameter (short-axis in lymph nodes), while other tumor manifestations could shrink or grow less than 20% in diameter [39]. Growth of non-measurable tumor manifestations and primary refractory disease were classified as diffuse progression. OPD lesions were retrospectively characterized as eligible for local treatment when they were radiologically amenable to surgery, radiotherapy, and/or percutaneous ablation and when the clinical condition of the patient permitted such a treatment.

For cases with clinical suspicion of alternative diagnoses, e.g., radiation pneumonitis or a second primary neoplasm, transbronchial or CT-guided percutaneous biopsies were performed for histological verification by the treating physicians. Cases without evidence of malignancy (or with diagnosis of a second primary tumor) in these biopsies were analyzed as not having disease progression. TTP was calculated from start of immunotherapy and compared between patient groups with the log-rank test. The cumulative incidence of OPD was calculated by considering OPD and diffuse progression as competing risks and compared among patient subgroups with Gray’s test. Time from OPD to first subsequent progression and overall survival from the start of immunotherapy were calculated according to Kaplan–Meier and compared with the log-rank test. Cox regression was performed for multivariable analysis of overall survival, and logistic regression was performed for multivariable analysis of factors associated with the development of OPD vs. diffuse progression. Categorical variables (like the presence of OPD) were compared among patient groups with the chi-squared test, and continuous variables were compared with the Student’s *t*-test. Duration of follow-up was calculated using the reverse Kaplan–Meier method. Statistical analyses were performed with SPSS (IBM, Armonk, NY, USA) and NCSS (Kaysville, UT, USA), while most figures were created with GraphPad Prism (La Jolla, CA, USA). The study was approved by the ethics committee of Heidelberg University (S-145/2017).

## 5. Conclusions

Our study provides real-world evidence that OPD occurs with a considerable frequency of 10%–20% in lung cancer under immunotherapy and is readily detectable, prognostically favorable, and amenable to local therapies. Our findings provide an additional opportunity to further improve patient outcome in NSCLC.

## Figures and Tables

**Table 1:** Characteristics of patients with disease progression in this study.

			IO Monotherapy-Treated Stage IV NSCLC Patients			IO+CHT-Treated NSCLC Patients		*p*-Value ^1^
			OPD (13%, n = 38)	Diffuse PD (n = 259)	*p*-Value ^1^	OPD (13%, n = 10)	Diffuse PD (n = 65)	
**Age (Median; SD)**			**63 (10)**	**64 (9)**	**ns**	64 (11)	65 (10)	ns
**Gender (% male)**			58	58	ns	40	58	ns
**Smoking Status (%) ^2^**		never smokers	0	10	*p* < 0.05	20	22	ns
		ex-smokers	61	53	ns	40	48	ns
		current smokers	39	37	ns	40	30	ns
**ECOG PS (%) ^3^**		0	47	41	ns	50	40	ns
		1	47	58	ns	50	59	ns
		2	5	2	ns	0	1	ns
**Histology (%)**		adenocarcinoma	68	63	ns	90	88	ns
		squamous cell carcinoma	29	31	ns	1	7	ns
		other (LCNEC, NOS, mixed)	3	6	ns	0	6	ns
**No. of Metastatic Sites at IO Start (Mean; SD)**			2.4 (1.2)	2.5 (1.4)	ns	1.1 (2.5)	1.8 (2.7)	ns
**PD-L1 IHC ^4^ (Average % of Positive Cells; SD)**			65 (33)	41 (36)	*p* < 0.001	17 (22)	18 (30)	ns
**LNR (Mean; SD)**			0.24 (0.11)	0.23 (0.46)	ns	0.21 (0.09)	0.17 (0.14)	ns
**IO Treatment**		first line	18	71	*p* < 0.05	10	65	
		second-and-beyond line	20	188	*p* < 0.05			
**TTP from IO Treatment Start in Months, Median**			9	2	*p* < 0.001			
		first-line patients	11	2	*p* < 0.001	4	4	ns
		second-and-beyond-line patients	5	2	*p* = 0.015			
**OS from IO Treatment Start in Months, Median (Mean)**			n.r. (26)	10 (13)	*p* < 0.001			
		first-line patients	n.r. (39)	14 (15)	*p* < 0.001	n.r.	n.r.	ns
		second-and-beyond-line patients	16	10	*p* < 0.05			

(O)PD: (oligo) progressive disease; SD: standard deviation; ns: not statistically significant; PS: performance status; LNR (lymphocyte-to-neutrophil ratio); no.: number; nr: not reached; TTP: time-to-progression; OS: overall survival. ^1^ Statistical comparisons were performed with a chi-squared test for categorical, with a *t*-test for numerical, and with the log-rank test for time-to-event data. ^2^ Data available for 293/297 cases. ^3^ Data available for 259/297 cases. ^4^ Data available for 226/297 cases.

**Table 2:** Frequency and anatomical distribution of oligoprogression in IO-treated NSCLC.

Location of OPD, no. (%) ^1^		IO monotherapy			1L IO + CHT ^1^	TTP of OPD
		All OPD ^1^ (*n* = 38, 13%)	1L IO OPD (*n* = 18, 20%)	2^+^L IO OPD (*n* = 20, 10%)	All OPD Cases (*n* = 10, 13%)	in Months, Median (IQR)
**Lymph Nodes**	All	16 (5%)	8 (9%)	8 (4%)	0 (0%)	8 (4–14)
	mediastinal	10	6	4		
	axillary, cervical	5	2	3		
	abdominal	2	1	2		
**Brain**		15 (5%)	3 (3%)	10 (5%)	3 (4%)	4 (2–11)
**Lung**	All	9 (3%)	5 (6%)	5 (2%)	2 (2%)	7 (3–15)
	primary tumor	4	1	3	2	
	lung metastasis	5	4	2	0	
**Adrenal Gland**		6 (2%)	1 (1%)	4 (2%)	2 (2%)	8 (5–13)
**Bone**		3 (1%)	1 (1%)	0	3 (4%)	4 (3–27)
**Liver**		2 (< 1%)	1 (1%)	1 (< 1%)	1 (2%)	12 (7–15)
**Skin/Soft Tissue**		1 (< 1%)	0	1 (< 1%)	0	9 (n/a)
**No. of Anatomic Sites, Average** (**SD**)**^2^**		1.4 (0.5)	1.1 (0.3) *	1.5 (0.6)	1.1 (0.3)	
**No. of Lesions, Average** (**SD**)		1.8 (1.2)	1.4 (0.8) *	2.3 (1.4)	1.6 (0.8)	
**TTP in Months, Median**		11	11 **	5	6	
					
		**IO monotherapy**			**1L IO + CHT**	
**All Patients with PD (no.)**		**all (297)**	**1L (89)**	**2^+^L (208)**	**all (85)**	
**FU, Median** (**IQR**)		15 (9–21)	13 (7–21)	15 (9–22)	7 (3–10)	

(O)PD: (oligo) progressive disease; 1L: first line; IO: immunotherapy; CHT: chemotherapy; no.: number; TTP: time-to-progression; IQR: interquartile range; SD: standard deviation; 2^+^L: second line and beyond; FU: follow-up from IO start; n/a: not applicable. ^1^ Percentages refer to the parent populations of 297 IO-treated (1L 89, 2^+^L 208) and 75 IO + CHT-treated patients (Table 1); in 10 IO-treated patients and 2 IO + CHT-treated patients, OPD occurred in 2 organs, therefore the percentages sum to >100%. ^2^ Each lymph-node station (e.g., right supraclavicular lymph nodes) and each organ (e.g., brain, left adrenal gland) was considered as one anatomic site. * *p* < 0.05 vs. 2^+^L IO-treated patients. ** *p* < 0.01 vs. 2^+^L OPD patients.

## Supplementary Materials

The following are available online at https://www.mdpi.com/2072-6694/12/4/1046/s1, Table S1: Characteristics of oligoprogressive cases in this study, Table S2: Occurrence of oligoprogression in immunotherapy-treated NSCLC according to patient characteristics, Table S3: Overall survival of immunotherapy-treated NSCLC according to the pattern of progression and other patient characteristics.
