# Supplementary material for: Oligoprogressive Non-Small-Cell Lung Cancer under Treatment with PD-(L)1 Inhibitors
Source: Cancers (Basel). 2020 Apr 23;12(4):1046. doi: 10.3390/cancers12041046 (PMC7226015; doi:10.3390/cancers12041046)
Supplement: Supplementary file 1 [file cancers-12-01046-s001.pdf]

Supplementary material

Oligoprogressive non-small-cell lung cancer under treatment with PD-(L)1 inhibitors

Stephan Rheinheimer, Claus-Peter Heussel, Philipp Mayer, Lena Gaissmaier, Farastuk Bozorgmehr, Hauke Winter, Felix J. Herth, Thomas Muley, Stephan Liersch, Helge Bischoff, Mark Kriegsmann, Rami. A. ElShafie, Albrecht Stenzinger, Michael Thomas, Hans-Ulrich Kauczor and Petros Christopoulos

Table S1. Characteristics of oligoprogressive cases in this study.

#	Age	Gender	Smoking status	ECOG PS	Histology	Metastatic Sites, nr.	PD-L1 TPS	LNR	Line IO	TTP	OPD site(s)	Tandem OPD
1	78	m	1	1	s	4	90	0,35	2	36	4, 3	
2	77	m	1	0	a	2	95	0,19	4	725	2, 3	yes
3	79	m	2	0	o	4	0	0,07	3	303	5	
4	77	m	2	0	s	1	100	0,21	1	254	5, 7	
5	77	f	1	0	a	2	90	0,21	1	167	2	
6	74	m	1	1	s	2	80	0,15	2	158	3	
7	75	m	1	1	s	2	10	0,20	2	53	2, 5	yes
8	72	m	1	0	a	2	40	0,26	1	126	1	
9	74	f	2	1	a	1	100	0,22	1	929	5	
10	71	f	2	1	a	1	n/a	0,34	3	370	2, 3	
11	74	f	1	1	a	2	75	0,22	1	401	5	
12	71	m	1	1	s	2	60	0,15	2	240	4	yes
13	72	m	1	0	s	3	10	0,12	1	461	1	
14	69	f	1	1	a	2	10	0,28	2	149	2, 5	
15	67	m	1	1	a	5	na	0,19	2	511	5	
16	66	m	1	0	a	1	15	0,19	2	55	2, 5	yes
17	67	f	1	1	s	2	80	0,30	2	71	3	yes
18	67	m	2	1	a	2	90	0,19	1	211	5	
19	62	m	2	1	a	3	positive	0,33	2	54	4, 3	
20	65	f	1	2	a	4	80	0,08	2	74	3	
21	64	m	1	0	s	3	80	0,23	1	403	3	
22	63	f	1	1	a	1	70	0,14	2	50	3, 7	
23	64	f	1	1	a	1	2	0,57	3	147	5	yes
24	61	f	1	2	a	2	n/a	0,45	2	367	3	yes
25	62	m	2	0	a	2	60	0,27	1	318	2	
26	62	m	2	0	s	4	60	0,47	1	531	4	yes
27	60	f	1	1	a	5	0	0,48	4	879	3	
28	58	m	1	0	a	3	70	0,04	3	152	5	yes
29	53	m	1	0	a	2	5	0,25	4	281	8	
30	55	m	2	0	a	3	60	n/a	1	807	2, 6	
31	54	f	1	1	a	2	80	0,16	1	343	5	yes
32	54	f	2	0	a	2	90	0,19	1	86	5	yes
33	50	f	2	0	a	2	positive	0,26	1	1341	5	
34	49	f	2	1	s	3	70	0,24	1	84	3	
35	48	m	2	0	a	2	80	0,29	1	335	3	
36	48	f	1	1	s	2	80	0,20	2	338	4	
37	46	m	2	0	a	1	80	0,19	1	1021	2	
38	39	m	2	0	a	5	100	0,26	1	854	5	
39	81	m	1	1	a	2	10	0,26	1*	235	4	
40	74	m	1	0	a	1	1	0,15	1*	293	2	
41	74	f	0	1	s	2	30	0,22	1*	94	3	
42	72	f	2	1	a	2	70	0,19	1*	180	2, 4	
43	68	m	1	5	a	1	40	0,16	1*	42	6	
44	60	f	1	1	a	2	5	0,17	1*	331	3	yes
45	58	f	2	0	a	3	0	0,13	1*	247	3	
46	57	m	2	1	a	4	10	0,11	1*	123	6	
47	47	f	0	0	a	4	2	0,45	1*	49	2,6	
48	46	f	2	0	a	4	0	0,20	1*	269	1	

m/f: male/female; smoking status 0: never smoker, 1: ex-smoker, 2: current smokers; histology a: adenocarcinoma; s: squamous-cell carcinoma; o: other (adenosquamous, NOS, LCNEC); n/a: not available; LNR: lymphocyte-to-neutrophil ratio; IO line 1*: chemoimmunotherapy; OPD site 1: liver, 2: lung, 3: brain. 4: adrenals, 5: lymph nodes, 6: bone; 7: other.

Characteristics for the 48 oligoprogressive (OPD) cases of non-small-cell lung cancer (NSCLC) under immunotherapy (IO) analyzed in this study (38 under IO-monotherapy, 10 under chemoimmunotherapy, please see Table 1 of the main manuscript).

Table S2. Occurrence of oligoprogression in immunotherapy-treated NSCLC according to patient characteristics.

**Patient characteristic**	**Association with occurrence of OPD (vs. diffuse PD)**		
	**HR**	**p-value**	**95% CI**
age at the time of IO treatment start	0.994	0.755	0.958–1.031
gender (male vs. female)	0.958	0.904	0.479–1.918
smoking status (never vs. ex- vs. current)	1.089	0.770	0.614–1.930
ECOG PS at IO start (0 vs. 1 vs. 2)	1.118	0.700	0.634–1.974
histology (ADC vs. SCC vs. other)	0.947	0.856	0.524–1.709
number of metastatic sites at IO start	0.95	0.673	0.749–1.205
**PD-L1 TPS by IHC**	**1.012**	**0.021**	**1.002–1.023**
Lymphocyte-to-neutrophil ratio at IO start	1.121	0.760	0.54–2.324
IO monotherapy vs. chemoimmunotherapy	1.299	0.613	0.472–3.580
treatment line at IO start (1 vs. 2+)	0.797	0.586	0.353–1.800

ADC: adenocarcinoma; SCC: squamous-cell carcinoma; other histology: NOS, LCNEC, adenosquamous; TPS: tumor proportion score; IHC: immunohistochemistry; HR: hazard ratio; 95% CI: 95% confidence interval; line at IO start 1: first, 2+: second and beyond.

Multivariable logistic regression for the association between development of oligoprogression (OPD) in non-small-cell lung cancer (NSCLC) patients treated with immunotherapy (IO), i.e. PD-(L)1 inhibitors as monotherapy or in combination with chemotherapy, and the baseline patient characteristics shown in Table 1 of the main manuscript. This analysis was based on the same population as the data of Table 1, which was the entire main study population (i.e., all patients with documented radiologic disease progression, n = 297 for IO monotherapy and n = 75 for chemoimmunotherapy, Table 1 and Figure 1). Statistically significant results are highlighted in bold.

Table S3. Overall survival of immunotherapy-treated NSCLC according to the pattern of progression and other patient characteristics.

**Patient characteristic**	**Association with OS from start of IO treatment**		
	**HR**	**p-value**	**95% CI**
age at the time of IO treatment start	1.004	0.684	0.985–1.023
gender (male vs. female)	1.157	0.426	0.808–1.657
smoking status (never vs. ex- vs. current)	0.974	0.849	0.738–1.284
**ECOG PS at IO start (0 vs. 1 vs. 2)**	**1.432**	**0.028**	**1.039–1.974**
histology (ADC vs. SCC vs. other)	1.110	0.450	0.847–1.456
**number of metastatic sites at IO start**	**1.156**	**0.010**	**1.035–1.292**
PD-L1 TPS by IHC	0.999	0.611	0.994–1.004
Lymphocyte-to-neutrophil ratio at IO start	0.948	0.839	0.566–1.589
IO monotherapy vs. chemoimmunotherapy	0.661	0.189	0.357–1.226
treatment line at IO start (1 vs. 2+)	1.278	0.261	0.833–1.959
**pattern of progression (OPD vs. diffuse)**	**0.341**	**0.001**	**0.186–0.625**

ADC: adenocarcinoma; SCC: squamous-cell carcinoma; other histology: NOS, LCNEC, adenosquamous; TPS: tumor proportion score; IHC: immunohistochemistry; HR: hazard ratio; 95% CI: 95% confidence interval; OPD: oligoprogression.

Cox regression of overall survival (OS) from immunotherapy (IO) start in relationship to the pattern of progression and other patient characteristics in the main study population (i.e., all patients with documented radiologic disease progression, n = 297 for IO monotherapy and n = 75 for chemoimmunotherapy, Table 1 and Figure 1). Statistically significant results are highlighted in bold.

© 2019 by the authors. Licensee MDPI, Basel, Switzerland. This article is an open access article distributed under the terms and conditions of the Creative Commons Attribution (CC BY) license (http://creativecommons.org/licenses/by/4.0/).
